# Apoptosis Related Human Wharton’s Jelly-Derived Stem Cells Differentiation into Osteoblasts, Chondrocytes, Adipocytes and Neural-like Cells—Complete Transcriptomic Assays

**DOI:** 10.3390/ijms241210023

**Published:** 2023-06-12

**Authors:** Katarzyna Stefańska, Lucie Nemcova, Małgorzata Blatkiewicz, Wojciech Pieńkowski, Marcin Ruciński, Maciej Zabel, Paul Mozdziak, Marzenna Podhorska-Okołów, Piotr Dzięgiel, Bartosz Kempisty

**Affiliations:** 1 Department of Histology and Embryology, Poznan University of Medical Sciences, 60-781 Poznan, Poland; 2 Cellivia 3 S.A., 61-623 Poznan, Poland; 3 Institute of Animal Physiology and Genetics of the Czech Academy of Sciences, 27721 Libechov, Czech Republic; 4 Division of Perinatology and Women’s Diseases, Poznan University of Medical Sciences, 60-535 Poznan, Poland; 5 Division of Histology and Embryology, Department of Human Morphology and Embryology, Wroclaw Medical University, 50-368 Wroclaw, Poland; 6 Division of Anatomy and Histology, University of Zielona Góra, 65-046 Zielona Góra, Poland; 7 Prestage Department of Poultry Sciences, North Carolina State University, Raleigh, NC 27695, USA; 8 Division of Ultrastructural Research, Department of Human Morphology and Embryology, Wroclaw Medical University, 50-368 Wroclaw, Poland; 9 Department of Veterinary Surgery, Institute of Veterinary Medicine, Nicolaus Copernicus University in Torun, 87-100 Torun, Poland; 10 Division of Anatomy, Department of Human Morphology and Embryology, Wroclaw Medical University, 50-368 Wroclaw, Poland; 11 Department of Obstetrics and Gynecology, University Hospital and Masaryk University, 60177 Brno, Czech Republic; 12 Physiology Graduate Faculty, North Carolina State University, Raleigh, NC 27695, USA

**Keywords:** Wharton’s jelly, mesenchymal stem cells, RNAseq, MSC, differentiation, apoptosis

## Abstract

Wharton’s jelly-derived mesenchymal stem cells (WJ-MSCs) exhibit multilineage differentiation potential, adhere to plastic, and express a specific set of surface markers—CD105, CD73, CD90. Although there are relatively well-established differentiation protocols for WJ-MSCs, the exact molecular mechanisms involved in their in vitro long-term culture and differentiation remain to be elucidated. In this study, the cells were isolated from Wharton’s jelly of umbilical cords obtained from healthy full-term deliveries, cultivated in vitro, and differentiated towards osteogenic, chondrogenic, adipogenic and neurogenic lineages. RNA samples were isolated after the differentiation regimen and analyzed using an RNA sequencing (RNAseq) assay, which led to the identification of differentially expressed genes belonging to apoptosis-related ontological groups. *ZBTB16* and *FOXO1* were upregulated in all differentiated groups as compared to controls, while *TGFA* was downregulated in all groups. In addition, several possible novel marker genes associated with the differentiation of WJ-MSCs were identified (e.g., *SEPTIN4*, *ITPR1*, *CNR1*, *BEX2*, *CD14*, *EDNRB*). The results of this study provide an insight into the molecular mechanisms involved in the long-term culture in vitro and four-lineage differentiation of WJ-MSCs, which is crucial to utilize WJ-MSCs in regenerative medicine.

## 1. Introduction

The incidence of autoimmune diseases is constantly increasing and some of them are untreatable, such as type I diabetes, multiple sclerosis and rheumatoid arthritis, creating a demand for the development of new therapies rather than depending on traditional ones. Stem cell research is rapidly evolving and might offer a new perspective in regenerative and reconstructive medicine [1]. The choice of tissues containing stem cells is vast; however, each has its limitations. Embryonic stem cells (ESCs), although pluripotent and possessing unlimited self-renewal ability, pose a risk of teratoma formation after transplantation. Furthermore, the acquisition of cells from embryos is ethically controversial [2]. Similarly, induced pluripotent stem cells (iPSCs) that are engineered from adult somatic cells may transform into neoplasms [3]. Adult stem cells, however, are considered the safer choice for transplantation due to their limited differentiation capability [4]. Adult stem cells have been isolated from various adult tissues, as well as from extraembryonic tissues, such as the Wharton’s jelly located in the umbilical cord [5].

The umbilical cord starts to develop at day 26 of gestation from the extraembryonic mesoderm or embryonic mesoderm and is responsible for bidirectional blood flow between the mother and the fetus [6,7]. The umbilical cord is covered with a simple epithelium of amniotic origin and contains three umbilical vessels, namely two arteries and one vein. The distinct compartments of the umbilical cord include the umbilical cord lining, subamniotic stroma, intervascular stroma, perivascular stroma and vessel, each containing stem cell populations with varied stemness properties [8,9].

The stromal tissue in the umbilical cord was called Wharton’s jelly after Tomas Wharton, who was the first to describe it in 1656. Wharton’s jelly is a mucoid connective tissue protecting the umbilical vessels from compression [10]. This tissue is abundant in the extracellular matrix (ECM) and is composed of glycosaminoglycans, mostly hyaluronic acid, and collagen fibers, while the elastic fibers are absent [11,12]. The stromal cells located in Wharton’s jelly resemble fibroblasts; however, Takechi et al. [13] revealed that these cells were expressing actin, non-muscle myosin and desmin typical for muscle cells; therefore, were considered as myofibroblasts. Nanaev et al. [14] demonstrated that the differentiation of stromal cells towards myofibroblasts occurs in a timely manner during pregnancy, and the most differentiated cells are in the proximity of umbilical vessels. The majority of the cells in Wharton’s jelly constitute myofibroblasts; however, mast cells are also present [15].

Myofibroblasts located in Wharton’s jelly exhibit the properties of mesenchymal stem cells (MSCs), which makes this tissue particularly relevant in terms of regenerative medicine. According to Wang et al. [16], the migration of hematopoietic stem cells and fetal MSCs occurs through the umbilical cord from the yolk sac and aorta-gonadal mesonephros to the placenta, and then to the fetal liver and bone marrow. As a result, some of these cells are trapped in Wharton’s jelly and change their properties due to the new environment. Another hypothesis is that the myofibroblasts in Wharton’s jelly are derived from mesenchyme, which is already in the matrix of the umbilical cord. The assumed role of these cells is to secrete the components of ECM for the protection of the umbilical vessels [9].

As stated by the International Society for Cellular Therapy (ISCT), the MSCs must adhere to plastic in standard culture conditions, differentiate towards chondroblasts, adipocytes and osteoblasts, and express CD105, CD73, CD90, while not expressing CD45, CD34, CD14 or CD11b, CD79α or CD19 and HLA-DR surface molecules [17]. In addition, the differentiation of MSCs towards the aforementioned lineages should be confirmed via histochemical staining, namely Alizarin Red or von Kossa staining for osteogenic differentiation, Oil Red O staining for adipogenic differentiation, and Alcian Blue staining for chondrogenic differentiation [17]. 

Wharton’s jelly-derived MSCs (WJ-MSCs), besides the aforementioned antigens, have demonstrated the expression of high levels of CD29, CD44, CD146, as well as markers of pluripotency, such as OCT-4, SOX-2, NANOG, SSEA-3 and SSEA-4 [18]. The differentiation of WJ-MSCs towards osteogenic, chondrogenic and adipogenic lineages has been conducted multiple times [19,20,21,22,23,24]. However, WJ-MSCs exhibit broader differentiation capacity, and they are able to transform into the cells of all three primary germ layers. Several authors have reported the differentiation of WJ-MSCs towards neurons and glia [25,26,27,28], cardiomyocytes [29], skeletal muscle [30], hepatocyte-like cells [31,32,33], retinal progenitor cells [34], germ-like cells [35], insulin-producing cells [36,37], endothelial cells [38], and endometrial cells [39]. 

Although there are relatively well-established differentiation protocols for WJ-MSCs, the exact molecular mechanisms involved in in vitro long-term culture and differentiation remain to be elucidated. A deeper understanding of these processes is of critical importance in order to utilize WJ-MSCs in regenerative medicine on a more regular basis. An important consideration is the fact that MSCs applied in vivo are exposed to an ischemic environment and nutrient deprivation, which may increase the risk of apoptosis, although it seems that the appropriate preconditioning of MSCs may alleviate that effect [40]. Next generation sequencing (RNA-seq) provides an opportunity to analyze the cellular transcriptome and discover its changes during the differentiation of WJ-MSCs. Hence, this study aims to identify apoptosis-related genes involved in the process of the in vitro differentiation of WJ-MSCs towards osteogenic, chondrogenic, adipogenic and neurogenic lineages.

## 2. Results

### 2.1. Morphological Analysis

**Figure 1:**
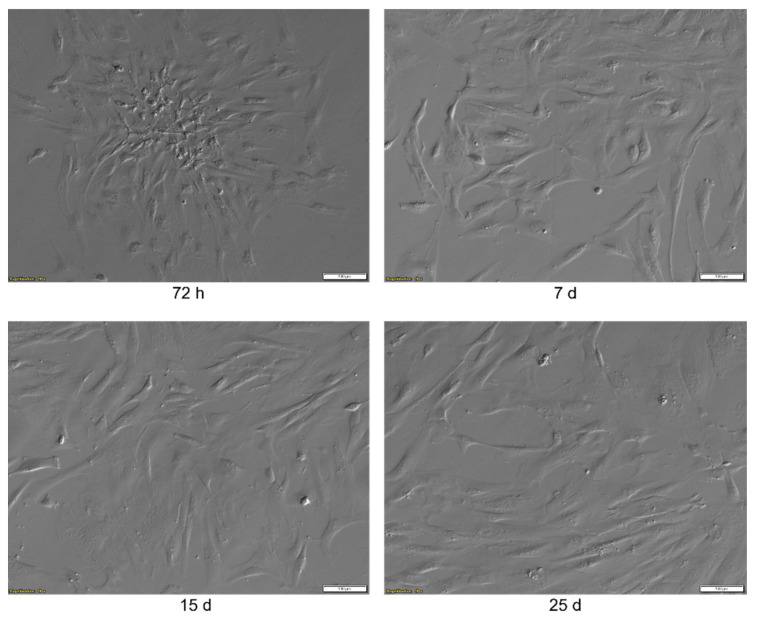
The results of the morphological analysis of the WJ-MSCs primary culture at 72 h, 7, 15 and 25 days. The pictures were taken at a 10× magnification. Scale bar: 100 µm.

The WJ-MSCs, after 72 h of primary culture, adhered to plastic and had accumulated in colonies where the cells exhibited a spindle shape, as it is presented in Figure 1. Subsequently, after 7 days of culture, the cells became more evenly distributed on the surface of the culture flask. Their shape was elongated as compared to cells after 72 h of culture. Their increase in size was also visible. After 15 days of culture, the WJ-MSCs became more flattened and densely packed, and this remained until day 25 of the culture and the subsequent subculture.

### 2.2. Flow Cytometry Analysis

Flow cytometry analysis was performed to confirm the MSC-like characteristics of the cells selected for further experiments. According to the analysis, the cells isolated from Wharton’s jelly exhibited the expression of markers typical for MSCs, namely CD105 (endoglin), CD73 (5′-nucleotidase) and CD44. In addition, the WJ-derived cells did not express CD31 (platelet endothelial cell adhesion molecule), CD34 and CD45 (protein tyrosine phosphatase receptor type C), which is consistent with the criteria that MSCs must fulfill. Therefore, the obtained results confirm that the cells isolated from Wharton’s jelly are the MSCs.

### 2.3. Evaluation of WJ-MSCs Differentiation

**Figure 2:**
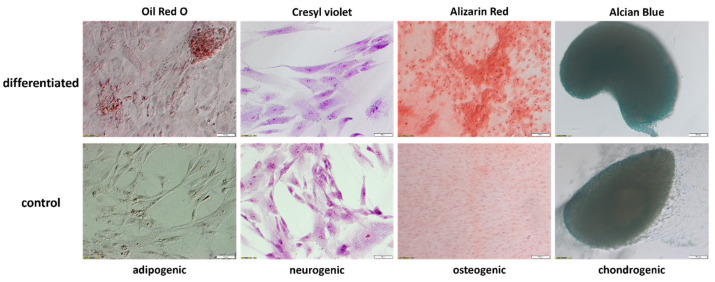
The results of Oil Red O, Cresyl violet, Alizarin Red, and Alcian Blue staining of the control and differentiated WJ-MSCs. The pictures of adipo- and neuro-induced WJ-MSCs were taken at a 20× magnification; scale bar: 50 µm, while the pictures of osteo- and chondro-induced WJ-MSCs were taken at a 10× magnification; scale bar: 100 µm.

WJ-MSCs after the third passage were differentiated into adipogenic, neurogenic, osteogenic and chondrogenic lineages. After a differentiation period, the cells were stained with Oil Red O for adipogenic differentiation, Cresyl violet for neurogenic differentiation, Alizarin Red for osteogenic differentiation, and Alcian Blue for chondrogenic differentiation. The results of the staining are presented in Figure 2. In the differentiated adipogenic samples, an intense red color could be observed, indicating the presence of lipid droplets; whereas, in the control sample, there was no presence of stain. Cresyl violet staining revealed the presence of Nissl bodies in the sample subjected to neurogenic differentiation and the lack of them in the control sample. After Alizarin Red staining, the differentiated sample exhibited an intense red coloring, indicating the presence of calcium deposits, which were not present in the control sample. Alcian Blue staining revealed an intense blue color in chondro-induced spheroids, indicative of a cartilage extracellular matrix, while the staining of the control spheroids was visibly less intense. Overall, the staining confirms the differentiation of the WJ-MSCs into adipocytes, neural-like cells, osteoblasts, and chondrocytes.

### 2.4. RNA-Seq Analysis

**Figure 3:**
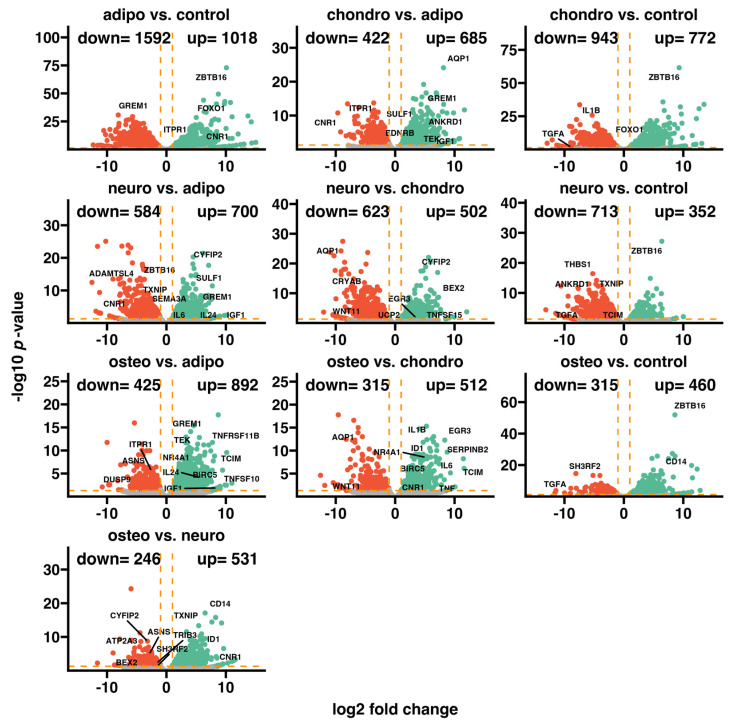
General expression profiles visualized as volcano plots, where each dot represents the mean expression of an individual gene. The orange dotted lines (cut-off values) were established according to the following parameters: |fold change| = 2 and *p*-value = 0.05. Genes above the cut-off lines were considered to be differentially expressed genes and are shown as red (downregulated) and green (upregulated) dots. The total numbers of up- and downregulated genes are provided in the top right and top left corners, respectively. The symbols of the five most differentially expressed genes from each composition are marked on the plots.

After the differentiation, we compared the whole transcriptome changes by using Bioconductor’s online packages. In the beginning, we analyzed the general expression profile of the transcriptome changes and presented it as volcano plots (Figure 3). With respect to the assumed cut-off criteria for the differentially expressed genes (|fold change| = 2, and *p* value < 0.05), we demonstrated 1018 upregulated (which was the highest number of overexpressed genes), and 1592 downregulated genes in the adipocytes vs. control. 

The comparison of the chondrocytes to the control revealed that 772 genes were upregulated and 943 genes were downregulated, while the neural-like cells vs. control indicated 352 upregulated and 713 downregulated genes. The comparison of the osteoblasts to the control indicated 460 upregulated genes and 315 downregulated genes, which was the lowest number across the whole analysis.

The top five genes mostly expressed in adipocytes compared to the control were *CNR1*, *ZBTB16*, *FRZB*, *FOXO1* and *ITPR1*. In the comparison of chondrocytes to the control, the list of genes with the highest expression profiles includes *ZBTB16*, *IGF1*, *WNT11*, *FOXO1* and *SEPTIN4*. Meanwhile, when we compared the neural-like cells to the controls, the most expressed genes were *ZBTB16*, *IGF1*, *BEX2*, *SEPTIN4* and *ITPR1*. In addition, a comparison of osteoblasts to the control revealed that *ZBTB16*, *SFRP2*, *CD14*, *EDNRB* and *TNF* were upregulated. In summary, we observed some similarities in the gene expression profile between the analyzed groups. The expression of *ZBTB16* and *FOXO1* genes was enhanced in osteo-, chondro-, adipo- and neuro-induced cells compared to the control, while *IGF1* was expressed highly in chondro-, neuro- and osteo-induced WJ-MSCs.

**Figure 4:**
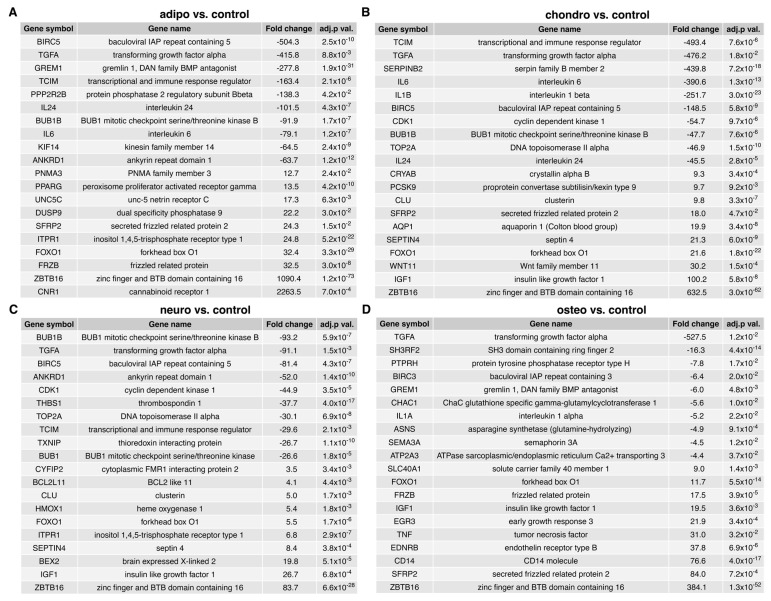
List of the top 20 genes with the highest (10 genes) and lowest (10) expression fold change between (**A**) adipocytes vs. control; (**B**) chondrocytes vs. control; (**C**) neural-like cells vs. control; and (**D**) osteoblasts vs. control. Abbreviations: adj. *p* val.—adjusted *p*-value.

**Figure 5:**
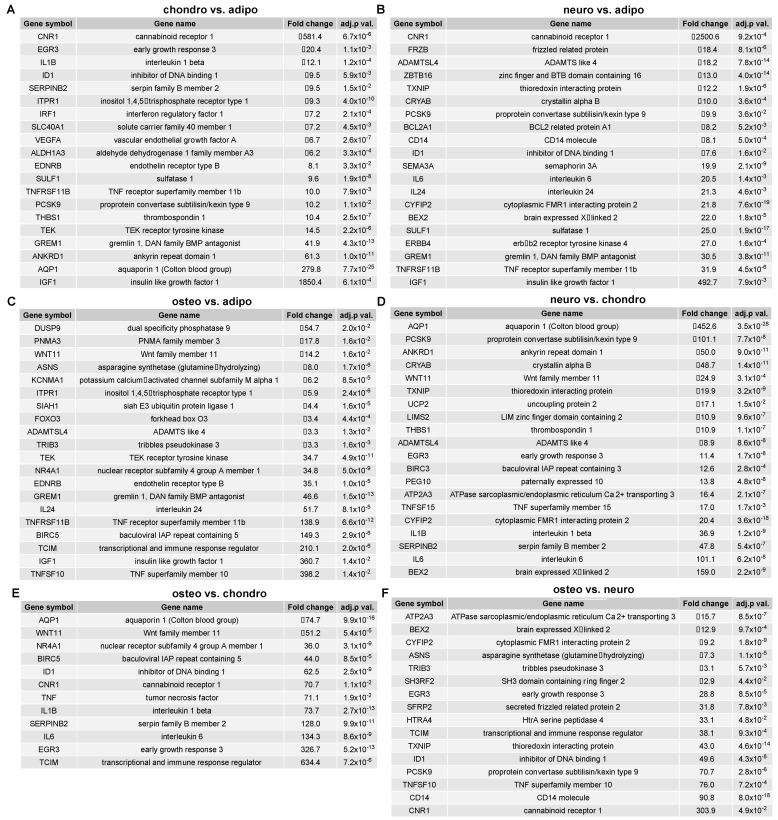
List of the genes with the highest and lowest expression fold change between all analyzed groups: (**A**) chondrocytes vs. adipocytes; (**B**) neural-like cells vs. chondrocytes; (**C**) osteoblasts vs. adipocytes; (**D**) neural-like cells vs. chondrocytes; (**E**) osteoblasts vs. chondrocytes; (**F**) osteoblasts vs. neural-like cells. Abbreviations: adj. *p* val.—adjusted *p*-value.

A list of the top 20 genes with the highest (10 genes) and lowest (10 genes) expression fold change in adipocytes, chondrocytes, neural-like cells and osteoblasts in contrast to the controls as well as a comparison between the groups is presented in Figure 4 and Figure 5.

The fold change values of the top ten downregulated genes in adipocytes vs. controls ranged from −504.3 to −63.7, while the expression of the top ten overexpressed genes ranged from 12.7 to 2263.5. The fold change values of the top ten downregulated genes in chondrocytes vs. controls ranged from −493.4 to −45.5, while the expression of the top ten overexpressed genes ranged from 9.3 to 632.5. Moreover, the fold change values for genes mostly downregulated in neural-like cells vs. controls ranged from −93.2 to −26.6, while the upregulated genes ranged from 3.5 to 83.7. For the osteoblasts to control comparison, the fold change for inhibited genes ranged from −527.5 to −4.4, and those of overexpressed genes ranged from 9.0 to 384.1. In conclusion, the commonly overexpressed genes in differentiated groups are *ZBTB16* and *FOXO1*, while *TGFA* was a downregulated gene in all differentiated cells. All genes are presented in Figure 4.

We also compared the differentially expressed genes between all differentiated cell groups (Figure 5. We revealed that in the adipocytes, enhanced expression was noticed for the *TNFRSF11B* (Fold changes vs. chondrocytes—10; vs. neural-like cells—31.9; vs. osteoblasts—138.9), *SULF1* (Fold changes vs. chondrocytes—9.6; vs. neural-like cells—25), *IL24* (Fold changes vs. neural-like cells—21.3; vs. osteoblasts—51.7), and *GREM1* (Fold changes vs. neural-like cells—30.5; vs. osteoblasts—46.6), genes. Moreover, in the group of chondrocytes, there appeared to be an upregulation of *IL6* (Fold changes vs. neural-like cells—101.1; vs. osteoblasts—134.3), *IL1B* (Fold changes vs. adipocytes—12.1; vs. neural-like cells—36.9; vs. osteoblasts—73.7), and *SERPINB2* (Fold changes vs. adipocytes—9.5; vs. neural-like cells—47.8; vs. osteoblasts—128) genes. Furthermore, the expression of the *TXNIP* gene (Fold changes vs. chondrocytes—19.9; vs. adipocytes—12.2; vs. osteoblasts—43) was upregulated in neural-like cells.

**Figure 6:**
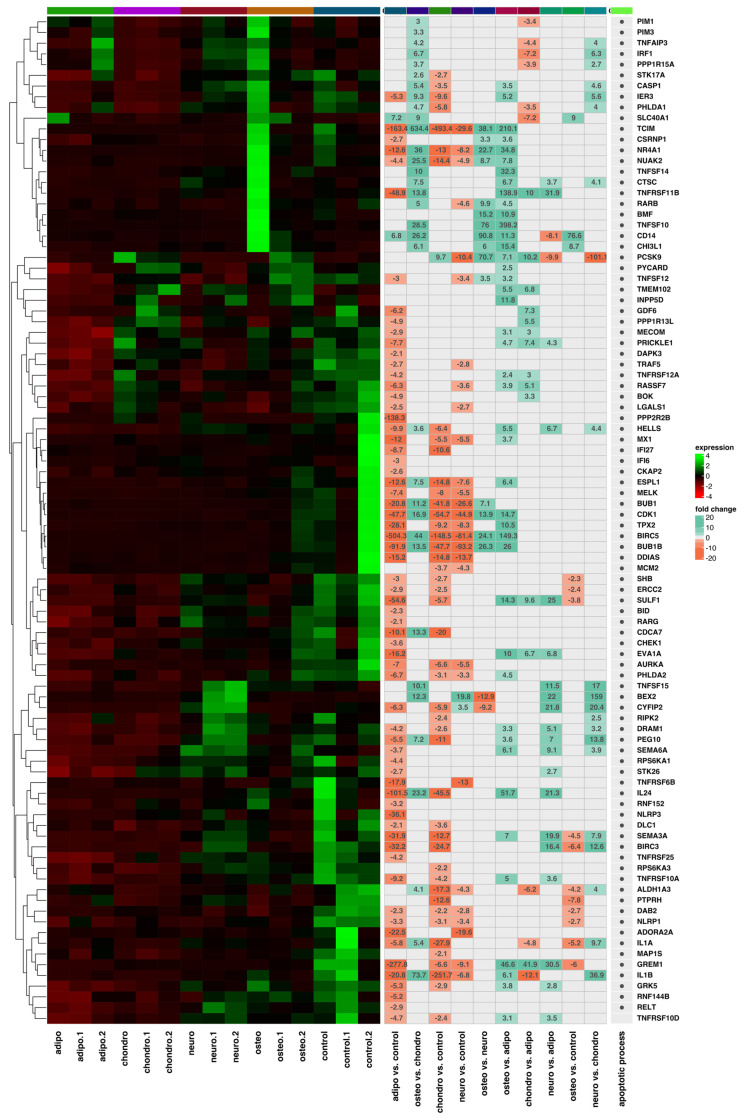
Heatmap with hierarchic clustering of differentially expressed genes related to the apoptotic process in all analyzed groups. Expression values are scaled by rows and presented as colours and range from red (low expression) to green (high expression).

**Figure 7:**
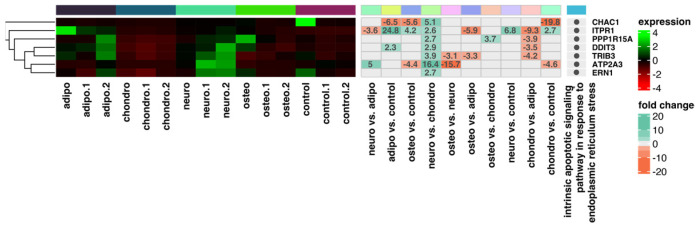
Heatmap with hierarchic clustering of differentially expressed genes related to the intrinsic apoptotic signaling pathway in response to endoplasmic reticulum stress in all analyzed groups. Expression values are scaled by rows and presented as colours and range from red (low expression) to green (high expression).

**Figure 8:**
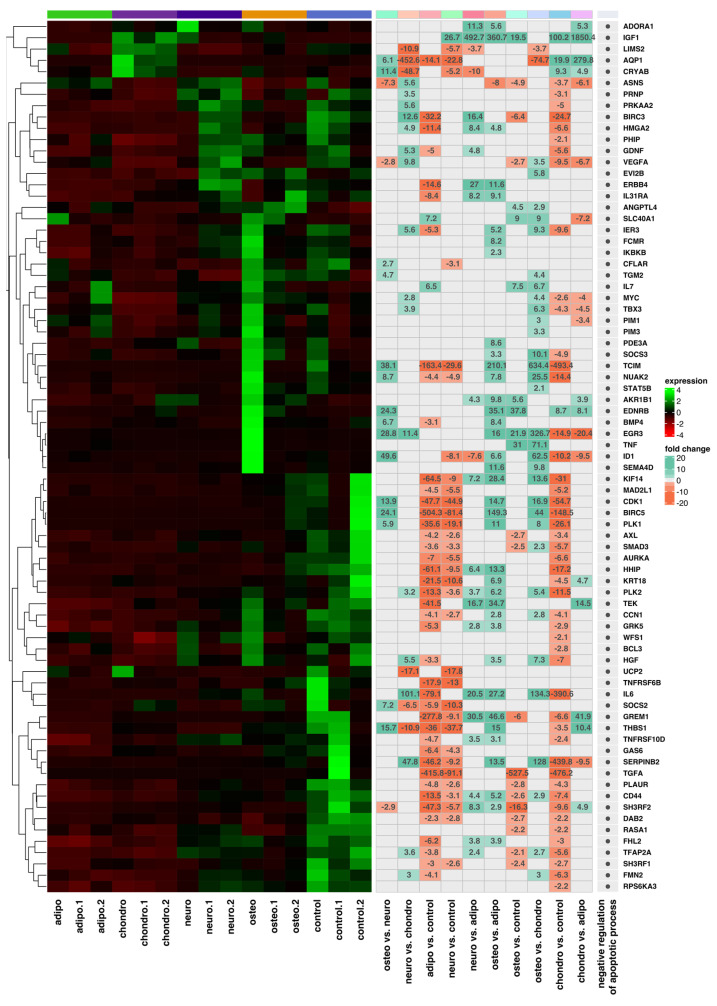
Heatmap with hierarchic clustering of differentially expressed genes related to the negative regulation of the apoptotic process in all analyzed groups. Expression values are scaled by rows and presented as colours and range from red (low expression) to green (high expression).

**Figure 9:**
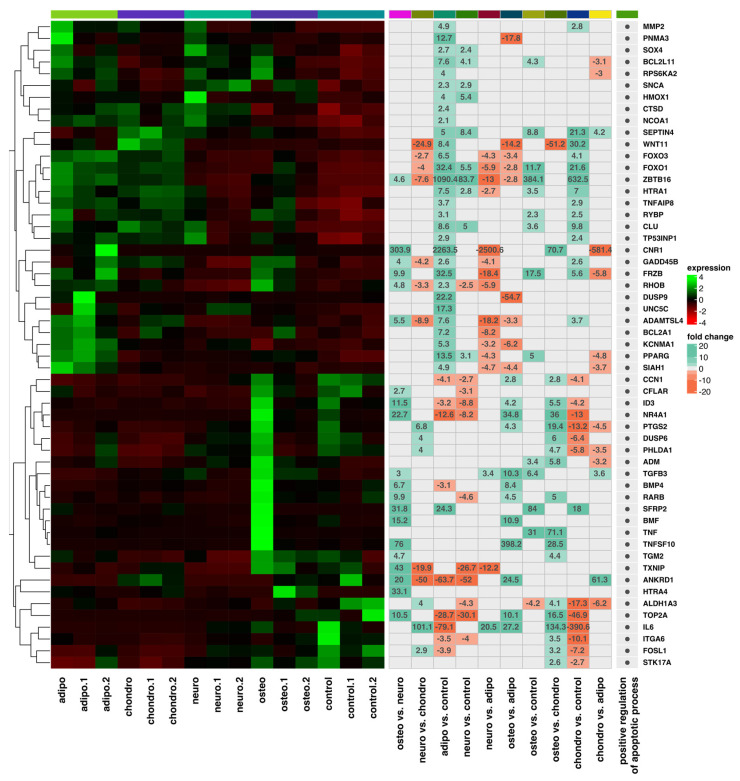
Heatmap with hierarchic clustering of differentially expressed genes involved in the positive regulation of the apoptotic process of all analyzed groups. Expression values are scaled by rows and presented as colours and range from red (low expression) to green (high expression).

As the next step, we performed a hierarchical clustering of differentially expressed genes in all analyzed groups and presented the results as heatmaps, which are presented in Figure 6, Figure 7, Figure 8 and Figure 9. The figure shows the mean expression values, normalized expression values, and fold changes between the compared groups. Genes that belong to the most significantly enriched ontological groups (with the lowest adjusted *p*-value) are represented as dark squares. The expression values were scaled by rows and presented as colors and ranges, wherein the fold changes were displayed in the rows. As a first step, we revealed which genes are involved in the apoptotic processes (Figure 6) depending on the differentiated cells, most of which were downregulated. In the adipocytes vs. controls, the genes most differentially expressed were *BIRC5*, *GREM1*, *TCIM*, *PPP2R2B* and *IL24*, all of which were downregulated. For the chondrocytes vs. controls, there was a downregulation of *TCIM*, *IL1B*, *BIRC5*, *CDK1*, and *BUB1B*. Meanwhile, at the neural-like cells, it appears that *BUB1B*, *BIRC5*, *CDK1*, *TCIM*, and *BUB1* were downregulated. Regarding the comparison of the osteoblast cells and control, it appears that apoptotic processes are involved through the expression of the *CD14*, *SLC40A1* and *CHI3L1* genes. *GREM1*, *NLRP1* and *DAB2* were the genes downregulated across all the studied groups compared to the controls. Furthermore, we analyzed the genes related to the apoptosis intrinsic apoptotic signaling pathway in response to endoplasmic reticulum stress (Figure 7). *ITPR1* was the only overexpressed gene in all the differentiated groups compared to controls. In the analysis of the genes related to the negative regulation of the apoptotic process (Figure 8), we found that the expression of *TGFA*, *GREM1*, *CD44*, *SH3RF1*, *DAB2*, *SH3RF2*, *PLAUR*, *SMAD3* and *AXL* was decreased in all differentiated groups as compared to the controls. Moreover, genes related to the positive regulation of the apoptotic process were clustered (Figure 9). These analyses reveal that the expression of the *ZBTB16*, *FOXO1*, *SEPTIN4*, *CLU* and *HTRA1* genes was enhanced in all analyzed groups compared to the controls. 

**Figure 10:**
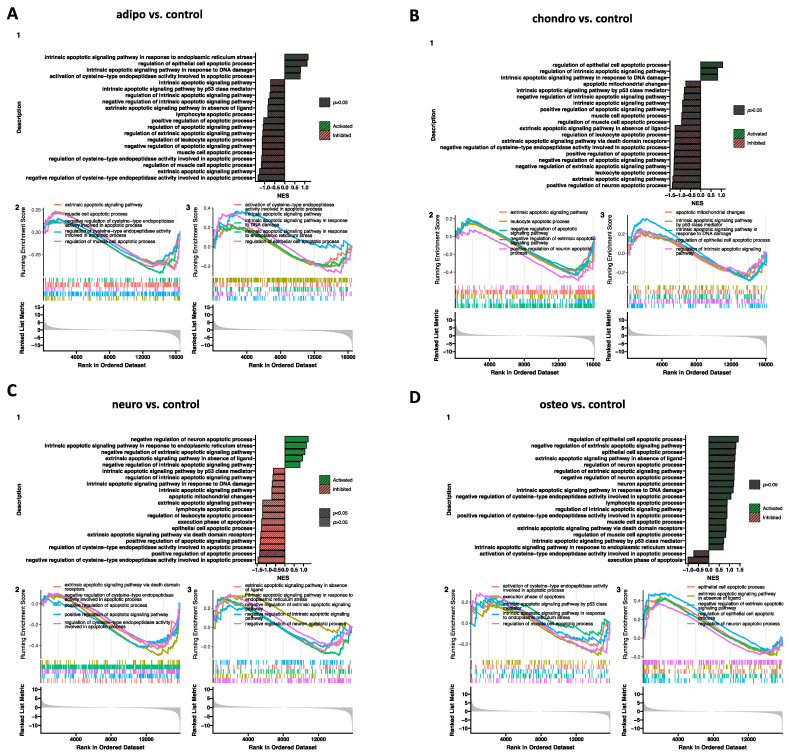
Gene set enrichment analysis (GSEA) for the comparison of the control to all analyzed groups; (**A**) adipo-induced WJ-MSCs vs. control; (**B**) chondro-induced WJ-MSCs vs. control; (**C**) neuro-induced WJ-MSCs vs. control; (**D**) osteo-induced WJ-MSCs vs. control. (1) Barplot with the most activated (green) and inhibited (red) gene terms according to the normalized enrichment score (NES) values. (2/3) Detailed enrichment plots for the five most inhibited/activated gene sets, showing the profile of the running ES score and the positions of the genes on the rank-ordered list.

**Figure 11:**
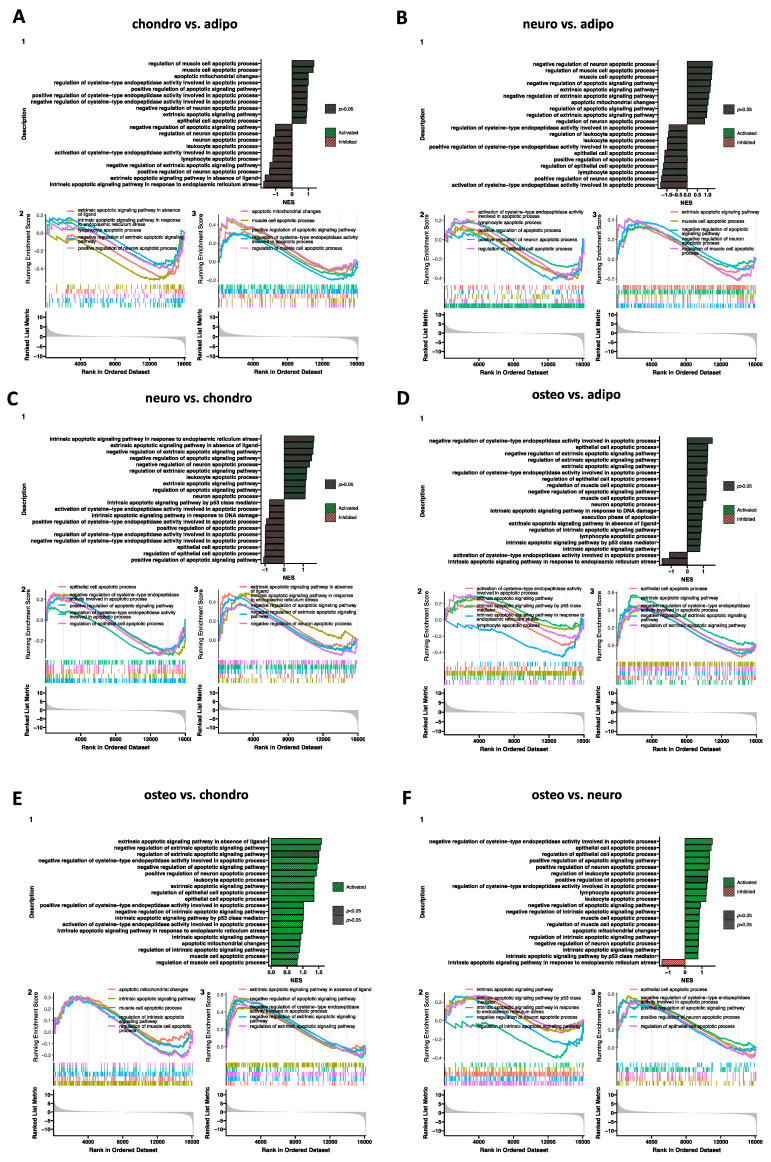
Gene set enrichment analysis (GSEA) for the comparison of all analyzed groups. (**A**) chondro-induced WJ-MSCs vs. adipo-induced WJ-MSCs; (**B**) neuro-induced WJ-MSCs vs. adipo-induced WJ-MSCs; (**C**) neuro-induced WJ-MSCs vs. chondro-induced WJ-MSCs; (**D**) osteo-induced WJ-MSCs vs. adipo-induced WJ-MSCs; (**E**) osteo-induced WJ-MSCs vs. chondro-induced WJ-MSCs; (**F**) osteo-induced WJ-MSCs vs. neuro-induced WJ-MSCs. (1) Barplot with the most activated (green) and inhibited (red) gene terms according to the normalized enrichment score (NES) values. (2/3) Detailed enrichment plots for the five most inhibited/activated gene sets, showing the profile of the running ES score and the positions of the genes on the rank-ordered list.

Additionally, a Gene Set Enrichment Analysis (GSEA) was performed to establish the received effects in all analyzed groups (Figure 10 and Figure 11). The normalized expression level data from the microarray were uploaded to the software, letting us generate a list of significantly described terms from the Hallmark database software. The GSEA did not indicate any statistical importances (*p* > 0.05). However, for some comparisons, such as neuro-induced vs. control, osteo-induced vs. chondro-induced, and osteo-induced vs. neuro-induced, we revealed that genes regulated in apoptotic processes are significantly activated (*p* < 0.05).

**Figure 12:**
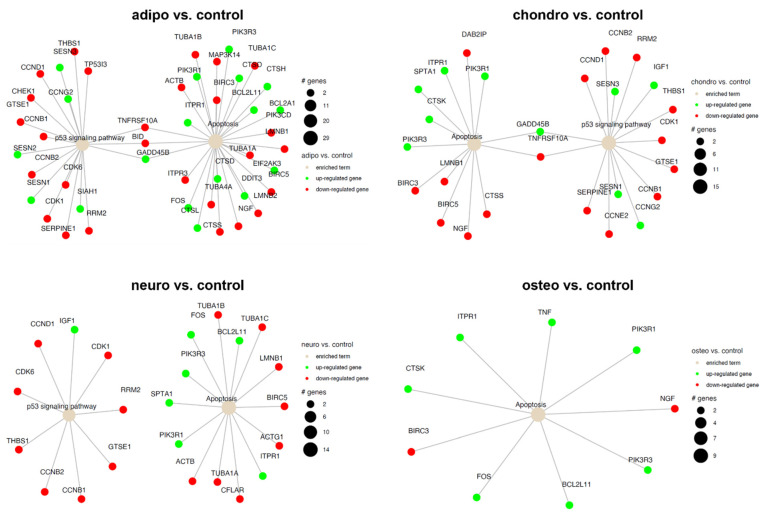
Apoptosis and p53 signaling pathway in the control compared to adipocytes, chondrocytes, neural-like cells and osteoblasts. Changes in the expression profile of genes involved in the pathway are marked in green for statistically significant upregulation and red for statistically significant downregulation. The beige color indicates the enriched term. The size of the bubble corresponds to the number of genes involved in a particular GO term.

**Figure 13:**
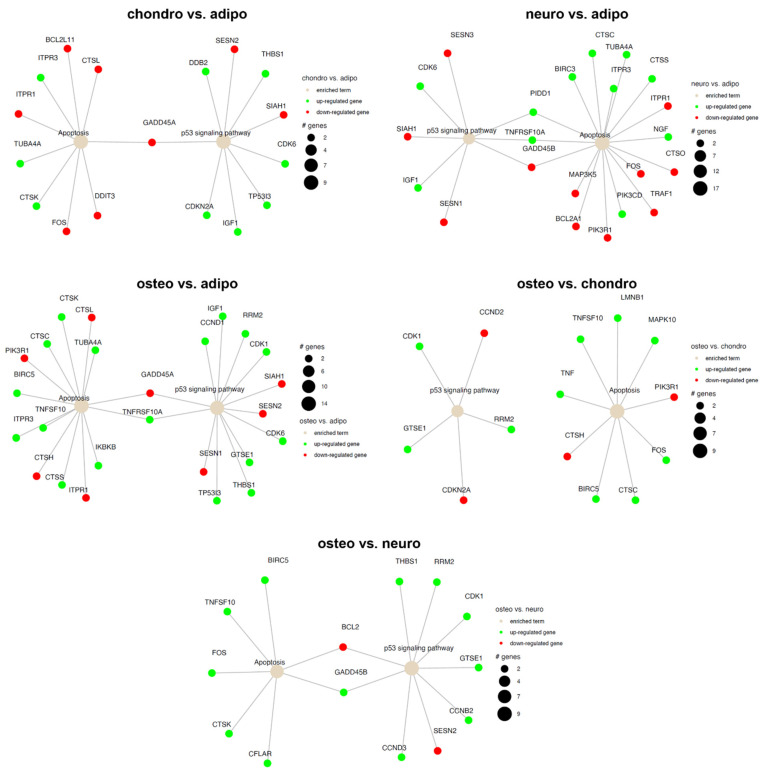
Apoptosis and p53 signaling pathway in all analyzed groups. Changes in the expression profile of genes involved in the pathway are marked in green for statistically significant upregulation and red for statistically significant downregulation. The beige color indicates the enriched term. The size of the bubble corresponds to the number of genes involved in a particular GO term.

The current data coincides with the well-documented association between apoptosis and the p53 signaling pathway (Figure 12 and Figure 13). The comparison of control and osteoblast cells did not indicate the expression of genes involved in the p53 signaling pathway (Figure 12). In the comparison of neural-like cells with chondrocytes, no expression of genes involved in the p53 signaling pathway or apoptosis was observed.

**Figure 14:**
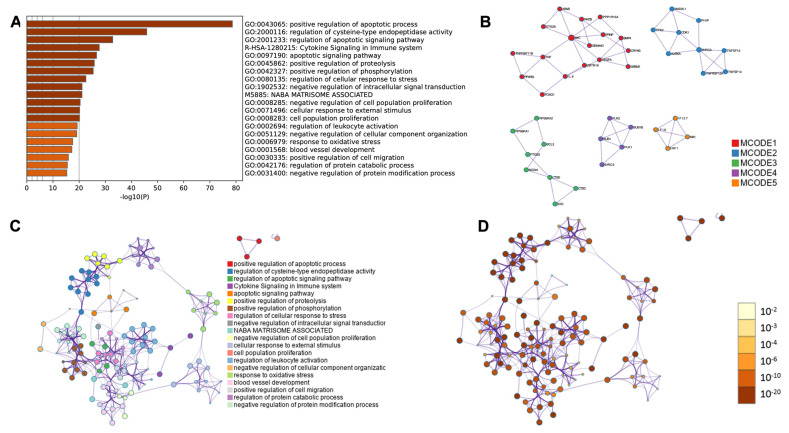
Transcriptome profiles were analyzed using Metascape functional analysis to identify the enriched Gene Ontology (GO) terms related to the apoptotic process, based on differentially expressed genes and GO BP terms. The results were visualized in four components. (**A**) A heatmap of enriched GO terms colored by *p*-values was generated; (**B**) The protein–protein interaction (PPI) network was clustered into the five most significant MCODE components, where each enriched GO term was represented by a circle node, with its size proportional to the number of input genes that fell under that term, and its color indicating its cluster identity; (**C**) A clustered network of enriched GO terms was created, where each term was represented by a circle node, with its size proportional to the number of input genes that fell under that term, and its color indicating its cluster identity; (**D**) A clustered network of enriched GO terms was generated, with the node colors indicating their *p*-values, and terms containing more genes having a more significant *p*-value.

Moreover, to conduct a comprehensive functional analysis of protein–protein interactions, a functional enrichment interactome analysis, gene annotation, and a membership search, we employed the online platform Metascape. For the analysis, we utilized four lists of differentially expressed genes that were categorized according to Gene Ontology biological process (GO BP) terms and obtained from heatmaps. We identified all the statistically enriched Gene Ontology (GO) terms, among which the top five enriched processes were: positive regulation of apoptotic process (GO:0043065; log10(P) = −78.6); regulation of cysteine-type endopeptidase activity (GO:2000116, log10(P) = −45.9); regulation of the apoptotic signaling pathway (GO:2001233, log10(P)= −32.9); cytokine signaling immune system (R-HAS-1280215, log10(P) = −27.7); and the apoptotic signaling pathway (GO:0097190, log10(P) = −26.7) (Figure 14A). A subset of representative terms was chosen from the entire cluster, converted into a network layout (Figure 14C,D), and analyzed using the MCODE algorithm to identify densely connected neighborhoods of proteins (Figure 14B).

## 3. Discussion

The aim of this study was to identify the apoptosis-related genes involved in the process of the in vitro differentiation of WJ-MSCs towards osteogenic, chondrogenic, adipogenic and neurogenic lineages utilizing RNA-seq. Microarray expression analysis may also be used for that purpose [41,42]; however, RNA-seq is a powerful technique used to analyze the transcriptomic changes occurring during the differentiation of WJ-MSCs. An RNA-seq allows for an in depth analysis of eukaryotic transcriptomes and the results are highly reproducible and might reveal sequence variations, as well as the most differentially expressed genes, providing possible markers of an investigated process [43,44]. Gaining an insight into the transcriptomic changes occurring during the in vitro differentiation of WJ-MSCs is of vital importance since in vitro cultures allow a better understanding of the molecular and cellular processes taking place in these cells [45]. This is particularly important when considering the use of WJ-MSCs in the clinical setting and most likely will contribute to the development of new treatment possibilities and therapies in regenerative medicine.

However, it is important to consider the fact that the in vivo application of MSCs is fraught with a high risk of cell death due to an ischemic environment and nutrient deprivation [40]. Potier et al. [40] revealed that after 120 h of hypoxia combined with serum deprivation, 99% of MSCs were not able to survive. Binder et al. [46] demonstrated, in the example of human BM-MSCs, that osteogenic differentiation promotes the survival of MSCs subjected to serum deprivation and hypoxia in vitro and in vivo, suggesting that the appropriate preconditioning of MSCs prior to their use for tissue regeneration may increase their efficacy. Similarly, Pesarini et al. [47] revealed that adipose tissue-derived MSCs (ASCs) were more sensitive to apoptosis caused by calcitriol combined with CaCl_2_ than ASCs subjected to adipogenic differentiation. Similarly, Lo Furno et al. [48] demonstrated a decrease in apoptotic markers in adipogenic-differentiated ASCs as compared to undifferentiated ASCs. On the contrary, Oliver et al. [49] showed that the in vitro adipogenic and osteogenic differentiation of human BM-MSCs was accompanied by an increased sensitivity towards apoptosis due to the decreased repair of DNA double-strand breaks.

Chondrogenically, adipogenically, neurogenically and osteogenically differentiated WJ-MSCs were related to each other and to WJ-MSCs not subjected to any differentiation regimen to search for the effects on the expression of apoptotic-related genes. Subsequently, a set of differentially expressed genes belonging to apoptosis-related ontological groups, namely “apoptotic process”, “intrinsic apoptotic signaling pathway in response to endoplasmic reticulum stress”, “negative regulation of apoptotic process”, and “positive regulation of apoptotic process”, was identified. 

*ZBTB16* (zinc finger and BTB domain containing 16) involved in the “positive regulation of apoptotic process” was upregulated in all differentiated cells as compared to the controls. ZBTB16 is a transcription factor that was already reported to be upregulated during the adipogenic, chondrogenic and osteogenic differentiation of MSCs [50,51,52,53,54,55]. In the case of neurogenic differentiation, the role of ZBTB16 is the least known. Sobieszczuk et al. [56] reported that ZBTB16 was involved in neuronal differentiation in Zebrafish, while Zhu et al. [57] showed the neuroprotective role of human umbilical cord-derived MSCs on spinal cord injury in mice, possibly due to ZBTB16, among others. Therefore, the current results correspond with previous studies and demonstrate, for the first time, the role of ZBTB16 in the four-lineage differentiation of WJ-MSC. Similarly, *FOXO1* (forkhead box O1) is involved in the “positive regulation of apoptotic process” and was one of the top five upregulated genes in WJ-MSCs subjected to chondrogenic and adipogenic differentiation and, in addition, one of the top ten genes upregulated in neuro- and osteo-induced WJ-MSCs. FOXO1 is a transcription factor participating in stemness and differentiation in several tissues [58]. Its role in the chondrogenic, osteogenic, adipogenic and neurogenic differentiation of MSCs has already been reported [59,60,61,62,63,64]. In terms of neurogenic differentiation, Dominguez-Castro et al. [65] reported the role of FOXO1 in WJ-MSCs specifically, revealing an elevated level of FOXO1 in WJ-MSCs during neuronal differentiation both in normoglycemic pregnancies and in pregestational diabetes mellitus. The current results are consistent with previous findings and provide evidence for FOXO1′s involvement in the in vitro differentiation of WJ-MSCs.

*SEPTIN4* is amongst the top five upregulated genes in chondro- and neuro-induced WJ-MSCs, it was also upregulated to a lesser extent in WJ-MSCs subjected to osteogenic and adipogenic differentiation. Similarly to the two previously described genes, it is involved in the “positive regulation of apoptotic process”, encoding the proapoptotic ARTS (apoptosis-related protein in the TGFβ signaling pathway). ARTS induces apoptosis [66,67]; however, its role in the differentiation of WJ-MSCs has not yet been described. The current results indicate its involvement in chondro-, neuro-, adipo- and osteo-induced WJ-MSCs, possibly via engaging in a proapoptotic function.

Amongst the top five genes upregulated in neuro- and chondro-induced WJ-MSCs was *IGF1* (insulin-like growth factor 1), which belongs to the “negative regulation of apoptotic process” group and encodes a protein generally involved in growth and development. The upregulation of *IGF1* in neuro-induced WJ-MSCs is consistent with its role in neurogenesis since it is associated with the enhanced proliferation and migration of neural stem cells, as well as with the inhibition of apoptosis and cell survival [68]. Moreover, a study conducted on umbilical cord-derived MSCs revealed that IGF1 could improve the neural differentiation of these cells and subsequent astrocyte differentiation [69]. The role of IGF1 in the chondrogenic differentiation of MSCs has already been reported. Zhou et al. [70] showed that IGF1 induced chondrogenic differentiation of ASCs in vitro and enhanced chondrogenesis in vivo. Furthermore, IGF1 was implicated in the osteogenic differentiation of MSCs [71], which coincides with the current results, as *IGF1* was amongst the top ten genes upregulated in osteo-induced WJ-MSCs.

*ITPR1* (inositol 1,4,5-triphosphate receptor type 1) belonging to the “intrinsic apoptotic signaling pathway in response to endoplasmic reticulum stress” group was upregulated in neuro- and adipo-induced WJ-MSCs. *ITPR1* encodes a receptor for inositol 1,4,5-triphosphate (IP_3_), which mediates Ca^2+^ release from the endoplasmic reticulum upon stimulation, and mutations in *ITPR1* are the cause of spinocerebellar ataxias [72]; thus, the role of ITPR1 in the nervous system is well-established. In the case of adipose tissue, ITPR1 has been associated with lipid accumulation and inflammation in preadipocytes, as well as with glucose homeostasis [73]. However, the role of ITPR1 in the neurogenic and adipogenic differentiations of WJ-MSCs was not yet described.

The other of the top five upregulated genes in adipo-induced WJ-MSCs include *CNR1* (cannabinoid receptor 1) and *FRZB* (frizzled related protein), which also belong to the “positive regulation of apoptotic process” group. Although the role of *CNR1* has not yet been described in the adipogenic differentiation of WJ-MSCs, Chen et al. [74] reported its upregulation in ASCs during osteogenic differentiation. In addition, *CNR1* is expressed in adipose tissue and might be involved in insulin resistance [75]. *FRZB* encodes SFRP3 (secreted frizzled-related protein 3), which is involved in the regulation of bone development. SFRP3 has been demonstrated to participate in the osteogenic and chondrogenic differentiation of BM-MSCs [76,77] and the adipogenic differentiation of ASCs [78], while the current study shows its involvement in the adipogenic differentiation of WJ-MSCs.

*WNT11* constitutes the last of the top five upregulated genes in chondro-induced WJ-MSCs. *WNT11* belongs to the “positive regulation of apoptotic process” group and has already been implicated in the chondrogenic differentiation of human MSCs; however, none of these cells were derived from Wharton’s jelly [79]. 

The remaining gene of the top five upregulated genes in neuro-induced WJ-MSCs has not yet been implicated in the neurogenic differentiation of these cells. *BEX2* (brain expressed x-linked 2), belonging to the “apoptotic process” ontology group is involved in broadly defined apoptosis. A protein encoded by *BEX2* was demonstrated to exert anti-apoptotic effects when overexpressed in breast cancer cells and malignant glioma cells [80,81]. Although *BEX2* is expressed in the central nervous system, its precise role in the neurogenic differentiation of MSCs remains unclear [82]. Thus, *BEX2* is a potential novel marker involved in the neurogenic differentiation of WJ-MSCs.

In osteo-induced WJ-MSCs, the top five upregulated genes include *SFRP2* (secreted frizzled related protein 2), *CD14* (CD14 molecule), *EDNRB* (endothelin receptor type B), and *TNF* (tumor necrosis factor), besides the aforementioned *ZBTB16*. Both *SFRP2* and *TNF* belong to the “positive regulation of apoptotic process” ontology group and were already implicated in the osteogenic differentiation of MSCs [83,84]. In addition, the overexpression of *SFRP2* in human MSCs has been demonstrated to enhance cell survival under oxidative stress [85]. The effect of TNF on the osteogenic differentiation of murine MSCs is dose-dependent [86]. In the case of MSCs derived from umbilical cords, TNF-α treatment was shown to induce osteogenic differentiation [87]. In turn, *CD14*, belonging to the “apoptotic process” ontology group, and *EDNRB,* implicated in the “negative regulation of apoptotic process”, were not directly associated with the osteogenic differentiation of MSCs. According to Dominici et al. [17], human MSCs should not express the CD14 molecule. CD14 has been shown to mediate the inflammatory response and rescue human monocytes from apoptosis [88]. In contrast, the overexpression of CD14 in gastric carcinoma cells has resulted in enhanced apoptosis and has antitumor potential [89]. *EDNRB* encodes a receptor for endothelin and its activation leads to cell proliferation and survival. Lee et al. [90] revealed that EDNRB participates in the regulation of lineage specification and its activation, due to the endothelin priming of BM-MSCs, was associated with the increase in osteogenesis of these cells. In addition, it was reported that neuropeptides may regulate the biological activity of the major bone cell types [91].

In summary, the upregulation of several genes involved in the apoptotic process was observed in all differentiated groups, indicating the importance of apoptosis-related genes in the four-lineage differentiation of WJ-MSCs. Several genes, such as *ZBTB16*, *FOXO1*, *IGF1*, *FRZB*, *WNT11*, *SFRP2* and *TNF* were already implicated in at least the one-lineage differentiation of MSCs; however, in most cases, these cells were not derived from Wharton’s jelly. Therefore, the current results confirm the role of these genes as the differentiation markers of WJ-MSCs as well. Moreover, potential novel markers of the osteogenic- (*CD14*, *EDNRB*, *SEPTIN4*), neurogenic- (*BEX2*, *ITPR1*, *SEPTIN4*), adipogenic- (*ITPR1*, *SEPTIN4*) and chondrogenic-differentiation (*SEPTIN4*) of WJ-MSCs were revealed. Overall, this study provides an insight into the molecular mechanisms involved in the in vitro long-term culture and differentiation of WJ-MSCs. It is important to uncover the effects of long-term in vitro culture and differentiation in the context of apoptosis prior to the clinical application of WJ-MSCs, considering the fact that MSCs applied in vivo may be fraught with the high risk of cell death due to the ischemic environment and a lack of nutrients. Since the current results indicate that most of the differentially expressed genes in WJ-MSCs subjected to four-lineage differentiation belong to the “positive regulation of apoptotic process” group, it should be considered whether prolonged in vitro culture and differentiation prior to clinical application is reasonable. Further studies are required to address this issue; however, based on the current results, the benefits of in vitro differentiation do not outweigh the flaws and the therapeutic application of WJ-MSCs should rather take place at the earlier stages of culture.

## 4. Materials and Methods

### 4.1. Material Collection

Samples of umbilical cord were obtained from healthy full-term deliveries with the written consent of the mother, according to the Ethics Committee of Poznan University of Medical Sciences (237/19). The age range of the patients was 24–40 years. The study was conducted according to the recommendations of the Declaration of Helsinki. Umbilical cords of around 15 cm length were collected in cold Dulbecco’s phosphate-buffered saline (DPBS; Merck, Darmstadt, Germany) with the addition of 10 U mL^−1^ penicillin, 10 mg mL^−1^ streptomycin and 25 µg mL^−1^ amphotericin B (Antibiotic Antimycotic Solution; Merck, Darmstadt, Germany), and transported directly to the laboratory within 24 h following acquisition.

### 4.2. Wharton’s Jelly-Derived Mesenchymal Stem Cells Isolation

The umbilical cords were washed twice in Dulbecco’s phosphate-buffered saline (DPBS; Merck, Darmstadt, Germany) with the addition of 10 U mL^−1^ penicillin, 10 mg mL^−1^ streptomycin and 25 µg mL^−1^ amphotericin B (Antibiotic Antimycotic Solution; Merck, Darmstadt, Germany) to remove residual blood. Then, the umbilical cords were placed on a Petri dish and sliced with the use of a sterile scalpel to 1 cm wide pieces. Furthermore, 2–3 mm pieces of Wharton’s jelly were excised from the umbilical cord’s tissue (excluding blood vessels and umbilical lining), with the use of sterile forceps. Obtained pieces of Wharton’s jelly were subsequently minced and incubated with 1 mg mL^−1^ collagenase type I (Gibco, Life Technologies, Waltham, MA, USA) for 24 h at 37 °C in a shaker. The cell suspension obtained after the digestion was centrifuged at 500× *g* for 20 min, and the supernatant was discarded. The cell pellet was suspended in DPBS and centrifuged at 500× *g* for 10 min. Then, the supernatant was discarded and the cell pellet was dissolved in 4 mL Dulbecco’s Modified Eagle’s medium (DMEM, Merck, Darmstadt, Germany), supplemented with 10% fetal bovine serum (FBS, Merck, Darmstadt, Germany), 4 mM of L-glutamine (Merck, Darmstadt, Germany), and 10 U mL^−1^ penicillin, 10 mg mL^−1^ streptomycin and 25 µg mL^−1^ amphotericin B (Antibiotic Antimycotic Solution; Merck, Darmstadt, Germany).

### 4.3. In Vitro Cell Culture

Cell viability was assessed using the ADAM Automatic Cell Counter (NanoEntek, Waltham, MA, USA) and only samples with more than 85% viability were used for primary cell culture establishment. The cell culture was conducted in 25 cm^3^ culture flasks at 37 °C in a humified atmosphere of 5% CO_2_. The culture medium was changed every 72 h. Cells were cultured until 90% confluent and then they were passaged using a 0.25% trypsin solution (Merck, Darmstadt, Germany). The primary in vitro culture was conducted until the third passage, and cellular morphology was evaluated daily using an inverted phase-contrast microscope (Olympus IX70, Olympus, Tokyo, Japan).

### 4.4. Flow Cytometry Analysis

During the third passage, half of the detached cells were subjected to flow cytometry analysis. Cells were incubated with the following antibodies: anti-CD44-PE, anti-CD90-FITC, anti-CD105-APC, anti-CD31-FITC, anti-CD73-PE, anti-CD45-PerCP, anti-CD34-PE, as well as the isotype controls: IgG1k-PE, IgG1-FITC, REA105-APC, REA-PE, IgG2ak-PerCP, IgG2ak-PE, IgG2ak-REA, for 30 min in darkness, according to the manufacturers’ protocols. Subsequently, the cells were washed with PBS (Merck, Darmstadt, Germany) and analyzed using the BD FACSAria™ cytometer (Becton Dickinson, Franklin Lanes, NJ, USA). 

### 4.5. Multilineage Differentiation

After the third passage, the cells were counted using the ADAM Automatic Cell Counter (NanoEntek, Waltham, MA, USA) and subjected to the osteogenic, neurogenic, chondrogenic and adipogenic differentiation regimen. Half of the culture plates were destined for RNA isolation, and half were destined for specific staining to confirm their differentiation.

#### 4.5.1. Osteogenic Differentiation

For osteogenic differentiation, the cells were seeded on 6-well culture plates at 1 × 10^5^ cells per well in standard culture medium. Each plate contained cells isolated from a separate umbilical cord. After the cells reached 100% confluency, the standard medium was replaced with Mesenchymal Stem Cell Osteogenic Differentiation Medium (PromoCell, Heidelberg, Germany) in half of the wells; whereas, in the remaining half, the cultures were conducted in a standard medium as negative controls. Differentiation was carried out for 14 days, with a medium change every 72 h. Then, the cells were washed with PBS, fixed with Saccomanno Fixative solution (Morphisto GmbH, Offenbach am Main, Germany) for 30 min, and stained with Alizarin Red S (Sigma-Aldrich, Saint Louis, MO, USA), which stains calcium deposits, in darkness for 15 min, according to the manufacturer’s protocol. The results of the staining were examined using an inverted phase-contrast microscope (Olympus IX70, Olympus, Tokyo, Japan).

#### 4.5.2. Neurogenic Differentiation

Neurogenic differentiation was conducted in 6-well culture plates. In total, 4 × 10^3^ cells/cm^2^ were seeded into single wells in a standard culture medium and cultured until 60–80% confluent, with the culture medium changed every 48 h. Then, the culture medium was replaced with Mesenchymal Stem Cell Neurogenic Differentiation Medium (PromoCell, Heidelberg, Germany) for seven days in half of the wells. The remaining wells contained cells cultured in a standard culture medium as negative controls. Differentiation results were examined with Nissl bodies staining. Briefly, the cell layer was washed with PBS and fixed with Saccomanno Fixative solution (Morphisto GmbH, Offenbach am Main, Germany) for 30 min at room temperature. Then, the cell layer was washed with PBS twice and stained with 0.5% Cresyl violet, previously filtered with the use of a 0.22 µm syringe filter (Millex, Merck, Germany), for 30 min at room temperature. Subsequently, the cell layer was washed three times with PBS and the results of the differentiation were examined using an inverted phase-contrast microscope (Olympus IX70, Olympus, Tokyo, Japan).

#### 4.5.3. Chondrogenic Differentiation

Chondrogenic differentiation was based on the spheroid model. For spheroid generation, the cells were seeded on a Nunc 96-well Round Bottom Microwell Plate (Thermo Scientific, Waltham, MA, USA) with 300,000 cells per well. The plates were incubated at 5% CO_2_ and 37 °C for 48 h, after which the cells had assembled into spheroids suitable for subsequent studies. After spheroid formation, the Mesenchymal Stem Cell Chondrogenic Differentiation Medium (PromoCell, Heidelberg, Germany) was added to half of the wells; whereas, in the other half, a standard culture medium was utilized for negative controls. The culture was conducted for 21 days, with a change of medium every 72 h. The results of the differentiation were evaluated with Alcian Blue (Sigma-Aldrich, Saint Louis, MO, USA) staining for aggrecan detection. Spheroids were washed gently with PBS and fixed with Saccomanno Fixative solution for 3 h at room temperature. Subsequently, the spheroids were washed twice with distilled water and stained with Alcian Blue, previously filtered with the use of a 0.22 µm syringe filter (Millex, Merck, Germany), for 45 min. The spheroids were washed three times with a destaining solution. The results of the staining were observed using an inverted phase-contrast microscope (Olympus IX70, Olympus, Tokyo, Japan).

#### 4.5.4. Adipogenic Differentiation

Adipogenic differentiation was conducted in 6-well culture plates. In total, 1 × 10^5^ cells per well were seeded in standard culture medium and cultured until 80–90% confluent. Then, the culture medium was replaced with Mesenchymal Stem Cell Adipogenic Differentiation Medium (PromoCell, Heidelberg, Germany) in half of the wells; whereas the other half contained cells cultured as negative controls in a standard culture medium. Differentiation was conducted for 14 days and the medium was changed every 72 h. The results of the differentiation were evaluated via Oil Red O (Sigma-Aldrich, Saint Louis, MO, USA) staining. The cell monolayer was washed with PBS and fixed with Saccomanno Fixative solution for 30 min at room temperature; then, the monolayer was washed with water and incubated with 60% isopropanol for 5 min. Subsequently, the cells were stained with Oil Red O for 3 min, and the results were observed using an inverted phase-contrast microscope (Olympus IX70, Olympus, Tokyo, Japan).

### 4.6. RNA Isolation

After differentiation, cells destined for RNA isolation (both the differentiated cells and controls) were detached using a 0.25% trypsin solution and suspended in 1 mL of TRIzol (Thermo-Fischer Scientific, Waltham, MA, USA) and immediately frozen at −80 °C. After phase separation using chloroform, total RNA was precipitated from the aqueous phase by adding isopropanol. Then, the total RNA was purified using an RNeasy Mini kit, eluted in 30 µL of RNAse/DNase free water, and stored at −80 °C after quality assessment. Quantification of the isolated RNA and its quality was performed using the Qubit™ RNA BR/HS Assay Kit and the Agilent RNA 6000 Nano/Pico Chip on the Bioanalyzer 2100 instrument, respectively. Both the concentration (6.2–335.0 ng/µL) and RIN values (6.9–10) met the criteria for library preparation.

### 4.7. RNA-Seq

A SMARter Stranded total RNA-Seq pico input Mammalian v3 kit was used for library preparation of the RNA samples with the input of 10 ng. Ribosomal RNA was depleted after cDNA synthesis and the library was amplified in 15 PCR cycles. The quantity (32.1–64.4 nM) of libraries passed the criteria for successful library preparation (more than 4 nM). Libraries were denaturated, diluted to final loading concentration (300 pM), and sequenced on a NovaSeq 6000 S4 flowcell with the aim of reaching 60M PE reads. A NovaSeq XP workflow was used for individual lane loading. Raw sequenced data were demultiplexed and QC metrics were generated. All the samples passed all the quality control parameters but noAdapters and low-quality sequences were trimmed using Cutadapt [92]. Trimmed raw reads were aligned to the human reference genome (hg19) from the Ensembl database. Alignment was performed using STAR software (version 2.5.2b) [93]. Overall summarization results, including the number of successfully assigned reads with unnormalized counts, were obtained using featureCounts [94]. Differential expression was determined using the Deseq2 library [95].

### 4.8. Bioinformatical and Statistical Analysis

Tabular data containing information about the fold change, adj. *p*.value, and the normalized counts for each comparison were analyzed using a BioConductor repository with the statistical R programming language (v4.1.2; R Core Team 2021). The selection criteria for differentially expressed genes (DEGs) were based on an absolute fold change > 2 and a *p*-value with a false discovery rate (FDR) correction < 0.05. The results of such selection were presented as volcano plots, illustrating the total number of up- and downregulated genes.

The complete set of DEGs from each comparison were subjected to functional annotation and clustering using the DAVID (Database for Annotation, Visualization, and Integrated Discovery) bioinformatics tool [96]. The gene symbols of DEGs were uploaded to DAVID using the “RDAVIDWebService” BioConductor library [97]. Then, we selected significantly enriched GO terms from the GO BP Direct database. The *p*-values of the selected GO terms were corrected using the Benjamini–Hochberg correction [98]. Hierarchic clustering of differentially expressed genes was performed, and the DEGs from each comparison were visualized as a heatmap using the “ComplexHeatmap” library [99].

Furthermore, Gene Set Enrichment Analysis (GSEA) has been performed by the “cluster profiler” library. The objective of this analysis was to determinate the extent of the depletion or enrichment in GO terms; thus, we limited the analysis only to GO terms related to apoptosis. A normalized enrichment score (NES) along with the corresponding *p*-value was calculated. To provide a summary of the most significant enrichment and depletion scores, a bar chart was created to display the ontology groups with the highest enrichment scores (highest NES values) as well as the groups with the most depleted enrichment scores (lowest NES values). Moreover, enrichment plots were generated for the five most enriched and depleted GO terms, offering a more detailed visualization of the enrichment levels. 

Next, we used the PathFinder library to identify and visualize the relationships between the DEGs and the biological pathways or processes in which they are involved [100]. We constructed a graph-based representation of the DEGs, where the edges correspond to genes and the central nodes correspond to selected biological processes between the gene expression levels. One of the key advantages of using PathFinder is the possibility of detecting relationships between genes and processes, which can be particularly useful in complex biological systems.

To identify functional protein partners among all the input gene lists, we utilized Metascape [101]. This database provides a comprehensive resource for the analysis and interpretation of gene and protein function, pathway analysis, and PPI network analysis. The minimum required interaction score was set at medium confidence (0.4). When the protein–protein interaction (PPI) network contained more than three nodes, the Detection (MCODE) algorithm was utilized to reveal clusters directly related to genes within the PPI [102]. Furthermore, MCODE assigned a unique color based on the *p*-value in the generated network.

## Data Availability

All of the data discussed in this work, if not already included in the manuscript, are available from the corresponding author on reasonable request.
